# The Discoverer of the Letheon

**Published:** 1847-06

**Authors:** 


### EDITORIAL DEPARTMENT.

*The Discoverer of the Letheon.*—We alluded, in our last number, to the fact that the credit of first applying the inhalation of the Vapor of Ether for the alleviation of pain in surgical operations, is being warmly contested by Drs. Jackson and Morton of Boston, in whose names, as joint discoverers, the *patent* was issued. Within a few days a friend has handed us a small pamphlet by Mr. Horace Wells, of Hartford, Conn., in which he claims that the application of the method originated with him. He states, that having made practical trials of the inhalation of the nitrous oxide gas in twelve or fifteen cases in which teeth were extracted without pain, he proceeded to Boston, and presented his discovery to several of the medical gentlemen of that city, among whom were Drs. Warren, Hayward, Jackson, and Morton. He, also, by invitation of Prof. Warren, at the close of one of his lectures, presented the subject before the class of medical students then in attendance at the Mass. Medical College; and, in connection therewith, submitted an experiment of extracting a tooth, which, not being very satisfactory, did not succeed in inspiring any confidence in the plan. This was in the autumn of 1844. He states that Dr. Morton was instructed in dentistry by himself, and that he was aware, at the period just mentioned, of his discovery. He alledges, farther, that Dr. Morton, at his request, applied to Dr. Jackson to prepare some nitrous oxide gas for the purpose, and that Dr. J. supplied him with the ether as being attended with the least trouble. Mr. Wells publishes affidavits from several individuals to the facts that he made successful experiments both with the nitrous oxide and ether, previously to the year 1844, and of his presenting the subject before the medical class of Boston as stated in his memoir.

We deem it but an act of justice to publish this condensed abstract of the pamphlet, more especially as the names of some of the gentlemen who have made affidavit to the statements contained therein are well known to the medical profession, and their testimony is entitled to full confidence. At the same time, we entertain a confident belief that the statements made by the different gentlemen involved in the controversy, which now appear to conflict with each other, are susceptible of some explanation which will spare any occasion for imputations of a want of honor or good faith on either side.

